# Characterization of pressure fluctuations within a controlled-diffusion blade boundary layer using the equilibrium wall-modelled LES

**DOI:** 10.1038/s41598-020-69671-y

**Published:** 2020-07-29

**Authors:** Radouan Boukharfane, Matteo Parsani, Julien Bodart

**Affiliations:** 1 Computer Electrical and Mathematical Science and Engineering Division (CEMSE), Extreme Computing Research Center (ECRC), King Abdullah University of Science and Technology (KAUST), Thuwal, 23955-6900 Saudi Arabia; 2 ISAE-Supaero, BP 54032, 31055 Toulouse Cedex 04, France

**Keywords:** Aerospace engineering, Mechanical engineering, Computational science

## Abstract

In this study, the generation of airfoil trailing edge broadband noise that arises from the interaction of turbulent boundary layer with the airfoil trailing edge is investigated. The primary objectives of this work are: (i) to apply a wall-modelled large-eddy simulation (WMLES) approach to predict the flow of air passing a controlled-diffusion blade, and (ii) to study the blade broadband noise that is generated from the interaction of a turbulent boundary layer with a lifting surface trailing edge. This study is carried out for two values of the Mach number, ${{\rm Ma}}_{\infty } = 0.3$ and 0.5, two values of the chord Reynolds number, ${{\rm Re}}=8.30 \times 10^5$ and $2.29 \times 10^6$, and two angles of attack, AoA $=4^\circ$ and $5^\circ$. To examine the influence of the grid resolution on aerodynamic and aeroacoustic quantities, we compare our results with experimental data available in the literature. We also compare our results with two in-house numerical solutions generated from two wall-resolved LES (WRLES) calculations, one of which has a DNS-like resolution. We show that WMLES accurately predicts the mean pressure coefficient distribution, velocity statistics (including the mean velocity), and the traces of Reynolds tensor components. Furthermore, we observe that the instantaneous flow structures computed by the WMLES resemble those found in the reference WMLES database, except near the leading edge region. Some of the differences observed in these structures are associated with tripping and the transition to a turbulence mechanism near the leading edge, which are significantly affected by the grid resolution. The aeroacoustic noise calculations indicate that the power spectral density profiles obtained using the WMLES compare well with the experimental data.

## Introduction

The noise generated by the flow of air passing over a lifting surface is a major issue for a wide range of engineering applications. Examples include the noise generated by propellers, rotors, wind turbines, fans, wings, and hydrofoils. Even in the absence of disturbances in the incoming stream, an airfoil can generate noise due to an unsteady turbulent boundary layer and wake, or through interactions with the lifting surface, particularly near the trailing edge region. This so-called self-noise or trailing edge noise is a major contributor to the overall noise generated by rotating machines and, in general, defines the lower bound of noise^1^. Reducing self-noise is challenging and requires an accurate and efficient method for describing and understanding the sources of noise near the trailing edge region. During recent decades, a variety of computational methods and theoretical models have been developed to predict the noise generated by unsteady flows passing over fixed or moving surfaces^2^. These approaches can be subdivided into three classes: semi-empirical, direct, and hybrid methods. The semi-empirical method, based on a set of acoustic data, is the most widely adopted for acoustic prediction when realistic aircraft designs must be used. However, the accuracy of the semi-empirical method is limited to standard flight conditions, and cannot be used for aircraft with unconventional airfoil profiles or operating conditions^3^. In contrast, the direct method uses a fully numerical method for both acoustic propagation and turbulence simulation and thus can provide accurate and reliable noise predictions^4^. However, these high-fidelity methods are prohibitively expensive and memory-intensive when used as a design and optimization tool. Hybrid methods offer an attractive compromise in terms of accuracy and computational cost because they combine (i) fluid dynamic calculations in the near field to resolve unsteady near-flowfields and (ii) the acoustic analogy for the propagation of sound to the far field^5^. Currently, the most popular method used for the aeroacoustic analogy is based on Ffowcs Williams and Hal’s seminal work^6^.

The power of model-based noise prediction depends on high spatial and temporal turbulent flow statistics, such as the length scales, spanwise and streamwise correlation length scales, degree of anisotropy, and acoustic spectral shape, which must be known a priori. large-eddy simulation (LES) is a promising high-fidelity approach that resolves the majority of energetic eddies in a turbulent flow while accounting for the effect of unresolved motions by subgrid scale (SGS) models^7,29^. Wall-resolved large-eddy simulation (WRLES) is extremely expensive because it must resolve the small but energetic structures in the near-wall region of the boundary layer. For instance, Terracol and Manoha^8^ employed 2.6 billion grid cells and six million core hours in their WRLES for a three-element airfoil with a chord Reynolds number of ${{\rm Re}}=1.23\times 10^6$. Because the small-scale energetic structures near the surface must be accurately captured, a large fraction of the cells in a typical computational domain are used to resolve the near-wall region of the boundary layer. Choi and Moin^9^ showed that the number of cells needed to resolve the inner layer (defined as $y^+=y u_\tau /\nu <100$, where *y* is the wall-normal direction) scales to ${{\rm Re}}^{13/7}$, which is a nearly quadratic dependence on the Reynolds number. The cost of a DNS calculation is even more prohibitive as the grid size scales to ${{\rm Re}}^{37/14}$^9^. A more affordable alternative to DNS and WRLES calculations is provided by the so-called wall-modeled LES (WMLES). This technique resolves large-scale flow structures in the outer region of the boundary layer only and models the effect of near-wall turbulence using a Reynolds-averaged Navier–Stokes (RANS) type model in the inner part of the boundary layer^10,11^. In contrast to detached eddy simulation (DES) methods^12^, which typically treat the entire attached boundary layer with a RANS approach, WMLES resolves most parts of the boundary layer with LES.

Bodart and Larsson^13^ used WMLES to achieve good agreement with the experimental data for the flow-field statistics of a McDonnell–Douglas 30P/30N multi-element airfoil at ${{\rm Re}}=9\times 10^6$ and estimated that the computational cost was reduced by two orders of magnitude compared to WRLES. Kawai and Larsson^14^ used a WMLES approach to simulate a supersonic flat-plate boundary layer at a Reynolds number based on the boundary layer thickness of ${{\rm Re}}_{\delta } = 6.1 \times 10^5$ and a free-stream Mach number of ${{\rm Ma}}_{\infty }=1.69$. They obtained a converged and predictive solution when compared with experimental results under the same flow conditions. George and Lele^15^ also performed WMLES to predict self-noise at a Reynolds number ranging from $1.0 \times 10^6$ to $1.5 \times 10^6$. In addition, they investigated the capability of the equilibrium WMLES^10^ to predict the flow around a wind turbine airfoil under stalled conditions, a NACA0012 airfoil in the near-stall regime, and a NACA64-618 airfoil in the post-stall regime.

All these applications using the WMLES approach highlight the potential of this emerging methodology for predicting aerodynamic and aeroacoustic fields and solving canonical problems. Less is known about using WMLES in complex flows, especially for the study of sound generation.

The present work has been carried out within the SCONE (Simulation of Contra Rotating Open Rotor and fan broadband NoisE with reduced order modeling) project^16^, which is part of the FP7 Clean Sky Joint Undertaking of the European Union that aims to investigate the noise generated by CROR/UHBR fan technologies and to achieve optimal noise reduction. Since it is difficult to experimentally measure flow pressure fluctuations on a surface without significantly affecting its motion, we instead employed a computational approach. Accurately predicting the airfoil self-noise generated by the interaction of a turbulent boundary layer with an airfoil trailing edge will provide enhanced inputs for current semi-analytic models. For this reason, the first objective of the SCONE project is to describe the flow over a controlled-diffusion blade with the ultimate aim to develop ‘quiet’ CROR and UHBR engines. The blade section geometry used here was originally part of an air-conditioning unit developed by Valeo that we slightly re-designed to impose a load on it similar to that of a CROR blade. We carried out the simulations for different values of Mach number (${{\rm Ma}}_{\infty } = 0.3$ and 0.5), Reynolds number (${{\rm Re}}=8.30 \times 10^5$ and $2.29 \times 10^6$), and angle of attack (AoA $=4^\circ$ and $5^\circ$). The configuration was a replica of the experiments performed within the CRORTET Clean Sky project, the computational results of which are compared to this study. Note that experimental aerodynamic data alone are not sufficient to provide the boundary layer parameters needed to normalize the wall-pressure spectra obtained in the experiment, which highlights the importance of LES for robust wall pressure modeling.

Numerical simulations of high Reynolds and Mach number flows around a controlled diffusion airfoil, which provide wall-pressure statistics, are scarce in the literature. However, to the best of our knowledge, convection velocity profiles and cross-spectra or coherence functions have not been analyzed in any previous studies that have used a WMLES approach. Therefore, our understanding of the multivariate statistics of the wall-pressure fluctuations induced by the interaction of a turbulent boundary layer and airfoil trailing edge in such flow conditions is still quite limited and thus needs to be improved.

This study represents a first step towards a deeper quantitative investigation of the effect of the Reynolds number, the angle of attack, and the upstream Mach number on the chord-wise development of the boundary layer characteristics of a controlled-diffusion blade. To this end, we use the compressible WMLES approach to compute the flowfield passing over a lifting surface. We validate the WMLES results against experimental data. In addition, we evaluate the efficacy of the WMLES approach for predicting the noise generated by a blade and investigate the source of noise and its generation mechanisms.

## Methods

### Governing equations

In this paper, the following notation is adopted: $x_1$ or *x* is the streamwise coordinate; $x_2$ or *y* is the wall-normal coordinate; and $x_3$ or *z* is the spanwise coordinate. The governing equations used in this study are the full Favre-filtered compressible Navier–Stokes which, for a calorically perfect gas, read

1 $$\begin{aligned}{\left\{ \begin{array}{ll} \frac{\partial{\overline{\rho }}}{\partial t}+ \frac{\partial{\overline{\rho }}\widetilde{{\mathscr{U}}_j}}{\partial x_j}=0, \\ \frac{\partial{\overline{\rho }}\widetilde{{\mathscr{U}}_i}}{\partial t}+ \frac{\partial \rho \widetilde{{\mathscr{U}}_j}\widetilde{{\mathscr{U}}_i}}{\partial x_j}+ \frac{\partial \overline{{\mathscr{P}}}}{\partial x_i}= \frac{\partial \breve{\tau }_{ij}}{\partial x_j}, \\ \frac{\partial{\overline{\rho }}\widetilde{{\mathscr{E}}}}{\partial t}+ \frac{\partial ({\overline{\rho }}\widetilde{{\mathscr{E}}}+ \overline{{\mathscr{P}}})\widetilde{{\mathscr{U}}_j}}{\partial x_j}= \frac{\partial \tau _{ij}\widetilde{{\mathscr{U}}_i}}{\partial x_j}- \frac{\partial \breve{{\mathscr{Q}}}_j}{\partial x_j}, \\ \overline{{\mathscr{P}}}={\overline{\rho }}{\rm R}\widetilde{{\mathscr{T}}}, \end{array}\right. } \end{aligned}$$

where $\rho$ is the density, ${\mathscr{U}}_i$ is the velocity component in the $x_i$-direction, ${\mathscr{P}}$ is the pressure, $\widetilde{{\mathscr{E}}}=\overline{{\mathscr{P}}}/[{\overline{\rho }}(\gamma -1)]+ \widetilde{{\mathscr{U}}_i}\widetilde{{\mathscr{U}}_i/2}$ is the total energy, ${\rm R}$ is the gas constant, ${\mathscr{T}}$ is the temperature, and $\gamma =c_{{\mathscr{P}}}/c_{\rm V}$ is the ratio of specific heats, which is kept constant at 1.4. The overline (resp. tilde) denotes the filtered (resp. Favre filtered) value. The variables are either spatially filtered or ensemble-averaged quantities depending on the use of LES or RANS equations. The latter model is used for the wall-model part. It is assumed that both the filtered stress tensor $\breve{\tau }_{ij}$ and the filtered heat flux $\breve{{\mathscr{Q}}}_j$ can be expressed in a way similar to their instantaneous counterparts, but applied to filtered quantities

2 $$\begin{aligned} \tau _{ij}=2(\mu +\mu _t)\widetilde{{\mathscr{S}}_{ij}}^{{\rm d}},\quad{\mathscr{Q}}_j=- (\lambda +\lambda _t)\frac{\partial \widetilde{{\mathscr{T}}}}{\partial x_j}, \end{aligned}$$

where $\mu$ is the molecular viscosity that has a power-law dependence on temperature

3 $$\begin{aligned} \mu =\mu _0(\widetilde{{\mathscr{T}}}/{\mathscr{T}}_0)^{0.76}. \end{aligned}$$

The parameter $\lambda$ in (2) is the molecular thermal conductivity, ${\rm Pr}=\mu c_{{\mathscr{P}}}/\lambda$ is the molecular Prandtl number kept constant at 0.7, and $\widetilde{{\mathscr{S}}_{ij}}^{{\rm d}}=\widetilde{{\mathscr{S}}_{ij}}- \delta _{ij}\widetilde{{\mathscr{S}}_{ij}}/3$ is the deviatoric part of the rate-of-strain tensor, which is defined as ${\mathscr{S}}_{ij}=(\partial \widetilde{{\mathscr{U}}}_i/\partial x_j+ \partial \widetilde{{\mathscr{U}}_j}/\partial x_i)/2$, where $\delta _{ij}$ is the Kronecker delta symbol. The other parameters $\mu _t$ and $\lambda _t$ in (2) are the turbulent eddy viscosity and conductivity, respectively. The LES solution is formally defined everywhere in the computational domain, and the wall-model equations are solved on a separate embedded grid near the wall. The only difference between the LES and the wall-model equations is in the computation of $\mu _t$ and $\lambda _t$. In the LES framework, $\mu _t=\mu _{{SGS}}$ and $\lambda _t=\lambda _{{SGS}}$ are the SGS eddy viscosity and conductivity, respectively. Note that the isotropic part of the modeled turbulent stress tensor is neglected in (2). This is often the case in the LES approach for low Mach number flows. The same assumption is made in the zero-equation RANS modeling context, but is less justified.

### Equilibrium wall-model

The equilibrium wall-model is derived from the compressible Reynolds-averaged Navier–Stokes equations with the boundary layer scaling approximations and neglecting unsteady and convective terms such as pressure gradient. The equilibrium model essentially relies on a grid resolution based on the outer layer requirements using the largest scales of the boundary layer thickness. The inner layer lies within the first cells normal to the wall and its behavior is modeled through the momentum wall normal flux. A typical resolution is about 20 cells per turbulent boundary layer thickness, $\delta$, in each spatial direction^10^. However, in this study, a fundamental question arises when considering the pressure fluctuations that are responsible for noise generation. In fact, to the best of our knowledge, there are no studies in the literature that guarantee that using a WMLES approach with a resolution of 20 cell per $\delta$ results in an accurate prediction of the turbulent structures responsible for the pressure fluctuations and hence, that can achieve an overall discretization that can predict the noise sources. However, because the location of the maximum pressure fluctuations is within the outer layer, this resolution appears to generate reasonable and satisfying results. The verification of this assumption is one of the primary goals of this paper.

In the following, we briefly review the equilibrium model used herein combined with the WMLES approach. Hereafter, we use the subscript “${{\rm wm}}$” to denote a quantity at the wall. Thus, given the instantaneous magnitude of the wall-parallel velocity ${\mathscr{U}}_{||}$ and the instantaneous temperature ${\mathscr{T}}$ in the LES at height $x_2=l_{{{\rm wm}}}$ perpendicular to the wall, we estimate the instantaneous wall shear stress vector $\tau _{w,i}$ by solving the following system using two ordinary differential equations (ODEs)^10^

4 $$\begin{aligned} \frac{d }{d x_2} \left[ \left( \mu + \mu _{t,{\rm wm}} \right) \frac{d{\mathscr{U}}_{||}}{d x_2}\right]= &{} 0, \end{aligned}$$

5 $$\begin{aligned} \frac{d }{d x_2} \left[ c_{\mathscr{P}}\left( \frac{\mu }{{\rm Pr}} + \frac{\mu _{t,{\rm wm}}}{{\rm Pr}_{t,{\rm wm}}}\right) \frac{d{\mathscr{T}}}{d x_2}\right]= &{} - \frac{d }{d x_2} \left[ \left( \mu + \mu _{t,{\rm wm}} \right){\mathscr{U}}\frac{d{\mathscr{U}}_{||}}{d x_2}\right] , \end{aligned}$$

where the wall-model eddy viscosity is taken as

6 $$\begin{aligned} \mu _{t,{\rm wm}} = \kappa \sqrt{\rho |\tau _w|} \, x_2 \left[ 1 - \exp \left( -\frac{x_2^+}{A^+}\right) \right] ^2. \end{aligned}$$

The values of the constants appearing (5) and (6) are $\kappa =0.41$, $A^+=17$ and ${\rm Pr}_{t,{\rm wm}}=0.9$. For compressible flows, the scaled distance from the wall in the Van Driest damping factor ($x_2^+$ in (6)) is computed using a semi-local scaling given by $x_2^+=x_2\sqrt{\rho |\tau _w|}$^17^. These two coupled ODEs are solved using the tridiagonal matrix or Thomas algorithm (TDMA) applied in a segregated manner, i.e., by alternating TDMA sweeps of the momentum and energy equations with updated eddy-viscosity $\mu _{t,{\rm wm}}$ in between. The pressure is assumed to be constant in the $x_2$ direction and is obtained from the LES solution at the exchange location, while the density is computed using the temperature via the equation of state.

One major difficulty in the parallel implementation of the model for unstructured grids is when the boundary condition for the wall-stress model (exchange location) and the wall location are located on different processors in terms of the domain decomposition associated with parallel computations. This issue is fixed by linking each wall face with its exchange location during the pre-processing step using parallel communicators, which are then used during the computation. This last point is important in terms of numerical errors that can arise within the first cell of the LES mesh. Indeed, these errors may significantly decrease the accuracy of the solution even though the model is correct. The recommended choice of the exchange location above the wall boundary is typically $\sim 0.1\delta$^14^. The grid spacing in the wall normal direction must be smaller than the resolution needed for fully turbulent simulations, which is a limitation of the WMLES approach for transitional flow. In fact, the typical wall normal grid spacings for WMLES based on turbulent boundary layer thickness, $\delta$, might be too coarse to resolve the pre-transitional laminar boundary layer and its instability. Park and Moin^18^ reported that, for WMLES in transitional cases, at least the size of the first grid cell near the wall $\Delta x_2^+<20$ is required to marginally resolve the integral length scales of the pressure-producing eddies near the wall. However, when using such a fine wall normal spacing, it may not always be possible to have the LES input taken at a distance even close to $\sim \, 0.1\delta$^19^. In the present study, because the flows are often transitional, the exchange location is taken at least three cells away from the wall, which lies at approximately 0.08–0.1$\delta$.

### Computational setup

The compressible Navier–Stokes equations are solved numerically using the massively parallel CharLES$^{{\rm X}}$ solver. This solver implements a cell-centered finite volume scheme that is minimally dissipative. The Euler flux is computed by a blend of a non-dissipative central scheme and a dissipative upwind scheme

7 $$\begin{aligned}{\mathfrak{F}} = \frac{1}{V} \int _{\partial V}{\mathbf{F}} \left({\mathbf{q}} \right) \cdot d{\mathbf{S}} = \sum _{f\in \text{faces}} (1-f_{\alpha f}){\mathfrak{F}}_{{CENTRAL}} + \, \, f_{\alpha f}{\mathfrak{F}}_{{UPWIND}}, \end{aligned}$$

where the blending parameter $0\le f_{\alpha f}\le$ is precomputed based on the local grid quality. To avoid numerical instabilities, the dissipative upwind-flux contribution is significant only in the region of relatively poor grid quality^20^. Indeed, the proportion of the upwind flux $f_{\alpha f}$ is not a static parameter, but it scales with the local departure of the global advection matrix (constructed with the central scheme) from a skew-symmetric matrix. For all the grids used in this study, the upwind proportion is less than 1.5% ($f_{\alpha f}<0.015$) in the regions with non-zero Reynolds stresses. Thus, the small-scale near-wall eddies and the large eddies passing through the separated shear layer, which are important in the dynamics of the separating and reattaching mechanisms, are largely unaffected by the numerical dissipation.

The numerical method is formally second-order accurate in space, although it achieves fourth-order accuracy on a uniform mesh containing only hexahedral cells. Time integration is performed using a third-order low-storage Rung–Kutta–Wray scheme^21^. Unresolved turbulent scales are modeled using the constant-coefficient Vreman subgrid scale model^22^.

**Figure 1:**
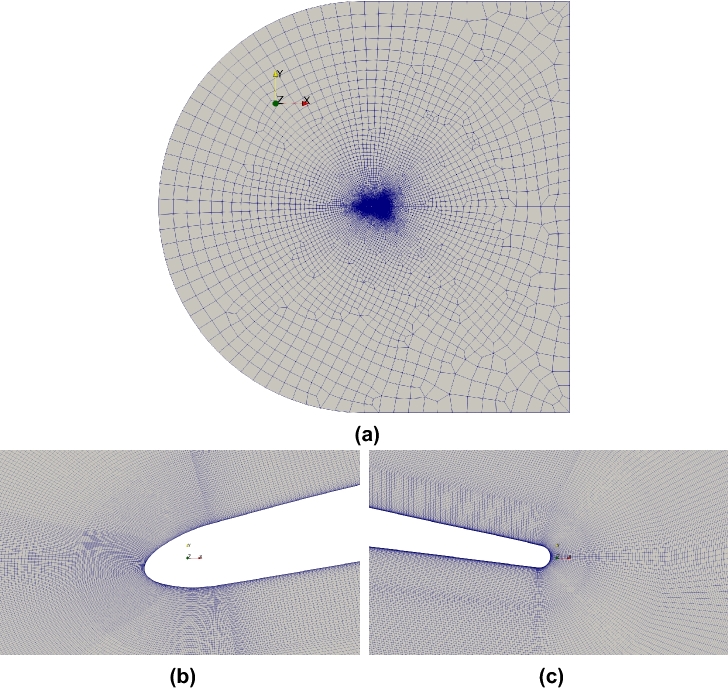
(**a**) Grid topology of the computational domain; WRLES mesh detail: (**b**) leading edge; (**c**) trailing edge and near wake.

The computational grid around the controlled-diffusion airfoil is topologically an O-type mesh due to the round leading and trailing edges, with boundary layer clustering at the airfoil walls. The boundary layer clustering blocks are structured blocks, whereas the rest are unstructured to allow for a quick outward coarsening. The computational grid is also equipped with wake blocks with a large angle of opening. Note that independently of the angle of attack to be simulated, the blocks are not rotated; hence, the mesh is not changed, as the inlet velocity angle is instead adapted. The computational domain extends 20*c* in both the streamwise, $x_1$, and wall-normal, $x_2$, directions, to allow for a velocity inlet boundary condition to act as free-stream (see Fig. 1). Unphysical numerical reflections at the computational boundaries are avoided by the choice of appropriate boundary conditions. Characteristic boundary conditions are used at all inflow and outflow boundary conditions^23,24^. At the airfoil surface, an adiabatic, no-slip condition is applied. Periodic boundary conditions are imposed in the spanwise direction.

Although there are no experimental data dealing with the spanwise correlation length, Grebert et al.^25^ found in their WRLES that a spanwise extent $L_{x_3}$ of at least $2\delta _{\max }$ showed reasonable agreement with experimental data for an accurate estimate of the correlation length $\ell _z$.

**Table 1:** Grid spacing, resolution on the blade surface, and description of the main parameters used in the computations.

	Case	${{\rm Ma}}_{\infty }$	${{\rm Re}}$	AoA	$L_{x_3}$	$N_{\text{cells}}$	Type of LES
${{\mathcal{C}}}_1$	${{\mathcal{C}}}_{1,{\mathfrak{c}}_1}$	0.3	$8.30\times 10^5$	$4^\circ$	$10\%~c$	225.1M	WRLES
	${{\mathcal{C}}}_{1,{\mathfrak{c}}_2}$	0.3	$8.30\times 10^5$	$4^\circ$	$10\%~c$	19.9M	WMLES
	${{\mathcal{C}}}_{1,{\mathfrak{c}}_3}$	0.3	$8.30\times 10^5$	$4^\circ$	$10\%~c$	19.9M	WRLES
${{\mathcal{C}}}_2$	${{\mathcal{C}}}_{2,{\mathfrak{c}}_1}$	0.5	$2.29\times 10^6$	$4^\circ$	$10\%~c$	225.5M	WRLES
	${{\mathcal{C}}}_{2,{\mathfrak{c}}_2}$	0.5	$2.29\times 10^6$	$4^\circ$	$10\%~c$	19.9M	WMLES
	${{\mathcal{C}}}_{2,{\mathfrak{c}}_3}$	0.5	$2.29\times 10^6$	$4^\circ$	$10\%~c$	19.9M	WRLES
${{\mathcal{C}}}_3$	${{\mathcal{C}}}_{3,{\mathfrak{c}}_1}$	0.5	$2.29\times 10^6$	$5^\circ$	$20\%~c$	451.0M	WRLES
	${{\mathcal{C}}}_{3,{\mathfrak{c}}_2}$	0.5	$2.29\times 10^6$	$5^\circ$	$10\%~c$	19.9M	WMLES
	${{\mathcal{C}}}_{3,{\mathfrak{c}}_3}$	0.5	$2.29\times 10^6$	$5^\circ$	$10\%~c$	19.9M	WRLES

Three flow conditions from the matrix of computation proposed by the SCONE project^16^ are studied here, and their details are summarized in Table 1. For ${{\mathcal{C}}}_{*,{\mathfrak{c}}_1}$ cases, the resolution level is very similar to the standard DNS  resolution criteria^26,27^; see Table 2. For these simulations, the mesh resolution quality was also verified by Pope’s criterion^27^, which monitors the quantity $IQ_k={\mathscr{K}}/({\mathscr{K}}+{\mathscr{K}}_{{SGS}})$, where ${\mathscr{K}}$ and ${\mathscr{K}}_{{SGS}}$ are the resolved and the SGS turbulent kinetic energies, respectively. On the one hand, the resolved turbulent kinetic energy is evaluated as ${\mathscr{K}}=({\mathscr{U}}_1^{'2}+{\mathscr{U}}_2^{'2}+{\mathscr{U}}_3^{'2})/2$, where ${\mathscr{U}}_i^{'}$ is the root-mean square (RMS) value of fluctuating part of the velocity component ${\mathscr{U}}_i$. On the other hand, the SGS turbulent kinetic energy is estimated by ${\mathscr{K}}_{{SGS}}=(\nu _t/(C_M\Delta ))^2$, where $\nu _t$ is the kinematic turbulent viscosity, $C_M$ a constant set to 0.069^28^, and $\Delta$ is estimated as the cubic root of the elements volume. For the grids indicated in Table 2, we found that $IQ_k\ge 0.94$ for the three WRLES cases, which means that the resolution level is close to that of a DNS mesh.

**Table 2:** Grid spacing and resolution along the blade surface and description of the main parameters used for the WRLES computations.

Case	$\Delta x^+$	$\Delta y^+$	$\Delta z^+$	$\Delta t$
${{\mathcal{C}}}_{1,{\mathfrak{c}}_1}$	$\le 28.01$	$\le 1.02$	13.21	$1.54\times 10^{-5}$
${{\mathcal{C}}}_{2,{\mathfrak{c}}_1}$	$\le 28.28$	$\le 1.12$	13.24	$2.68\times 10^{-5}$
${{\mathcal{C}}}_{3,{\mathfrak{c}}_1}$	$\le 26.86$	$\le 1.22$	14.23	$2.88\times 10^{-5}$

**Figure 2:**
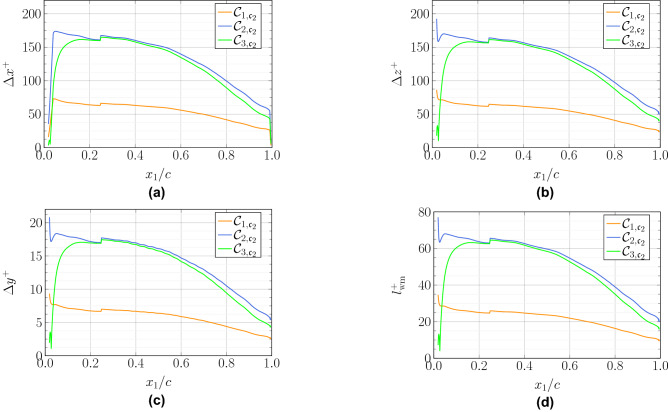
Grid spacing in wall units along the upper surface of the airfoil for the WMLES cases.

With the wall-model configuration, no near-wall streaks can be captured. In fact, the friction momentum flux is imposed by the model. Therefore, there is no need to follow any resolution requirement imposed by these structures. Note that when considering the $x_2$ direction, for example, the number of points remain essentially unchanged compared to WRLES. Instead, the first cell close to the wall increases in size. This ensures a proper resolution in the outer layer and drastically increases the time step because of the Courant–Friedrichs–Lewy (CFL) limit, which is directly dependent on this length scale. The overall cost ratio between the WRLES and the WMLES simulations is close to 50. The grid resolution used for the WMLES is chosen to resolve the flow scales in the outer layer and thus, the grid spacing is scaled by the local boundary layer thickness. As a consequence, the mesh does not strongly depend on the Reynolds number. As indicated in Table 2, the same grid is used for all WMLES computations. The cases labeled with ${{\mathcal{C}}}_{\star ,{\mathfrak{c}}_3}$ all have the same grid resolution but the wall-model is not activated to investigate its effect at an iso-mesh resolution. The grid distributions in wall units for these computations along the chord are shown in Fig. 2. The grid sizes for all the WMLES cases have $\Delta x_1\approx \Delta x_3$. In order to capture the boundary layer transition on the upper surface of the blade and achieve as smooth a flow as possible at the airfoil trailing edge, the grid spacing in the wall normal direction is fine, i.e., $\Delta y^+$ is less than 20 for the three cases. To allow errors due to the subgrid modeling and numerics to be made arbitrarily small, as shown in the study by Kawai and Larsson^14^, five grid points ($l_{\text{wm}}=y_5$) off the wall in the LES mesh are matched to the wall-model top boundary in this work. The variations of the height of the exchange location in viscous units are included in Fig. 2d. In the present study, the local wall-model layer thickness is set to a maximum of 4 times the local wall-normal grid-spacing and a user-defined minimum thickness $l_{\text{ud}}$. The effects of the exchange location on the flow field, especially on the velocity profiles, will be investigated in future simulations.

Finally, it is important to emphasize that for the WMLES calculations, the non-dimensional time-step size $\Delta t=\Delta t^{\star }{\mathscr{U}}_{\infty }/c$ (where the superscripts $^{\star }$ denote dimensional quantities) is larger due to the coarse grid resolution near the wall, and approximately two orders of magnitude bigger than that used for the WRLES computations. Furthermore, the maximum CFL number is $\sim \, 0.4$.

## Results

In this section, we simultaneously: (i) investigate the generation of airfoil trailing edge broadband noise that arises from the interaction of a turbulent boundary layer with an airfoil trailing edge and (ii) study the performance of the equilibrium boundary layer wall-model presented in “Equilibrium wall-model”. Describing a flow as it passes a controlled-diffusion blade is challenging because it involves complex physical processes: laminar separation, turbulent transition, turbulent reattachment, and turbulent separation near the trailing edge on the suction side. We compare our numerical results with the experimental database generated within the framework of the CRORTET Clean Sky project^29^.

### Flow topology

**Figure 3:**
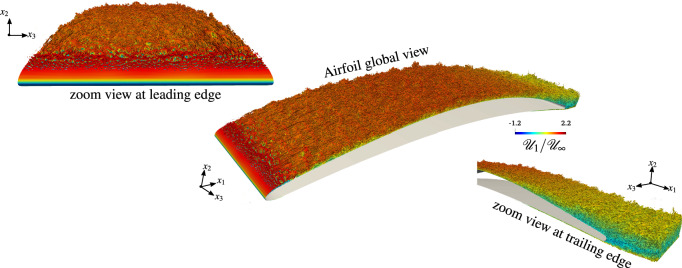
Topology of the flow described by the *Q*-criterion ($Qc^2/{\mathscr{U}}_\infty ^2\sim 950$) colored by the normalized longitudinal instantaneous velocity ${\mathscr{U}}_1/{\mathscr{U}}_\infty$ for the ${{\mathcal{C}}}_{3,{\mathfrak{c}}_1}$ case for which ${{\rm Ma}}_\infty =0.5$, ${{\rm Re}}=2.29\times 10^6$ and AoA $=5^\circ$.

The three-dimensional features of vortex structures and their breakdown processes in the laminar-turbulent transition region near the leading edge are visualized by the instantaneous iso-surfaces of the second invariant of velocity gradient tensor, $Q=(\omega ^2+2{\mathscr{S}}_{ij}{\mathscr{S}}_{ij})/4$, in Fig. 3. The *Q* iso-surfaces are colored by streamwise velocity to approximately identify the height of these vortex structures. According to the definition of *Q*-criterion, a vortical structure is identified in a region with positive *Q*, i.e, a region where vorticity overcomes the strain.

A fully laminar boundary layer is present on the lower (pressure) side up to the trailing edge of the airfoil, as well as a transitional and turbulent boundary layer on the upper (suction) side. A transition from a laminar to a turbulent state occurs in the shear layer resulting from the flow separation in the leading edge region, which results in the massive generation of vorticity downstream of the leading edge, with large vortices shed from the suction side of the airfoil. However, some smaller vortices still remain attached to the wall and roll over the airfoil suction side, grazing the trailing edge. As the curvature of the controlled-diffusion airfoil changes, the adverse pressure gradient leads to an increase of the boundary layer thickness. At the trailing edge, the laminar boundary layer coming from the pressure side destabilizes into a small vortex-shedding. This Von Kármán street then interacts with the fully turbulent vortical structures issuing from the upper side.

**Figure 4:**
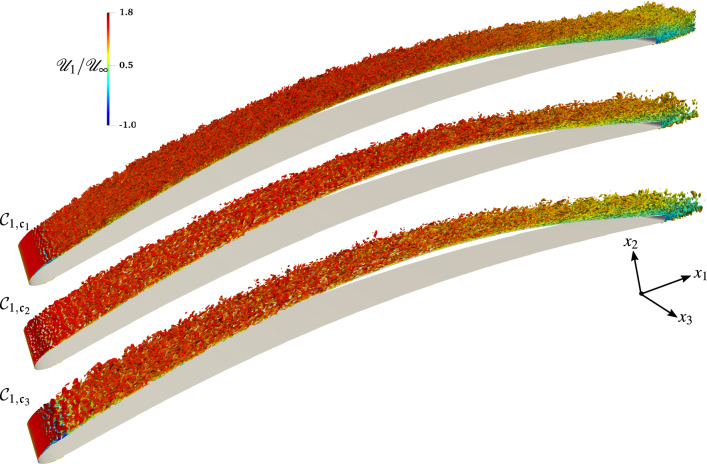
Topology of the flow described by the *Q*-criterion ($Qc^2/{\mathscr{U}}_\infty ^2\sim 950$) and colored by the normalized longitudinal instantaneous velocity ${\mathscr{U}}_1/{\mathscr{U}}_\infty$ for the ${{\mathcal{C}}}_{1}$ cases for which ${{\rm Ma}}_\infty =0.3$, ${{\rm Re}}=8.30\times 10^5$ and ${AoA}=4$.

**Figure 5:**
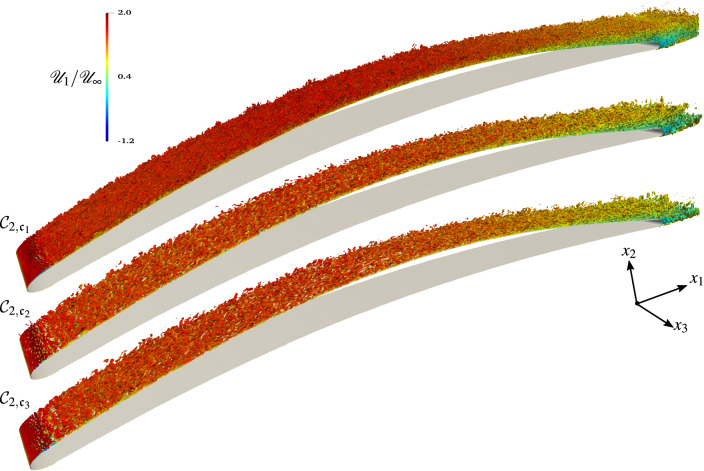
Topology of the flow described by the *Q*-criterion ($Qc^2/{\mathscr{U}}_\infty ^2\sim 950$) and colored by the normalized longitudinal instantaneous velocity ${\mathscr{U}}_1/{\mathscr{U}}_\infty$ for the ${{\mathcal{C}}}_{2}$ cases for which ${{\rm Ma}}_\infty =0.5$, ${{\rm Re}}=2.29\times 10^6$ and ${AoA}=4$.

**Figure 6:**
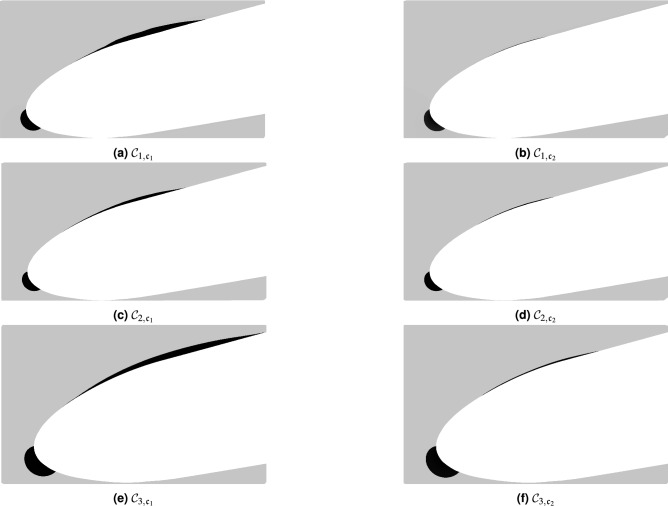
Visualization of the mean flow velocity sign for WRLES cases (left column) and WMLES cases (right column). Negative regions in black highlight the leading edge bubble.

Figure 4 depicts the level of vorticity and the size of turbulent structures in the flow at the same instant for all three ${{\mathcal{C}}}_1$ simulations. We observe that the vorticity and the vortex shedding is more intense for the ${{\mathcal{C}}}_{1,{\mathfrak{c}}_1}$ case (i.e., the WRLES) than for ${{\mathcal{C}}}_{1,{\mathfrak{c}}_2}$ and ${{\mathcal{C}}}_{1,{\mathfrak{c}}_3}$ cases. Furthermore, in the ${{\mathcal{C}}}_{1,{\mathfrak{c}}_1}$ case, many more structures are resolved. The nature of the developed turbulent structures is broadly influenced by the size and the structure of the recirculation bubble near the leading edge (see Fig. 4). Furthermore, the WMLES (i.e., the ${{\mathcal{C}}}_{1,{\mathfrak{c}}_2}$ case) does not show a laminar separation, and the flow becomes turbulent without the clear $2{{\rm D}}$ vortex breakdown, as is shown in the ${{\mathcal{C}}}_{1,{\mathfrak{c}}_1}$ and ${{\mathcal{C}}}_{1,{\mathfrak{c}}_3}$ cases. Indeed, the vortices are first generated very close to the wall due to the flow instability induced by the sudden increase in the wall shear stress $\tau _w$ (which is fed into the LES as flux boundary conditions) by activating $\mu _{t,{\rm wm}}$ in the wall-model at the leading edge. As shown in Fig. 5, by increasing both the Reynolds and the Mach numbers, the flow topology observed in the former case ${{\mathcal{C}}}_1$ is also reproduced in the ${{\mathcal{C}}}_2$ case, and we can see that, even when the flow seems to be similar at $x_1/c>0.2$, the processes that reach the turbulent state are completely different. The comparison of the WMLES results with the reference WRLES configuration (the ${{\mathcal{C}}}_{2,{\mathfrak{c}}_1}$ case) indicates that the equilibrium dynamic wall-model does not adequately capture the formation of two-dimensional intermittent laminar separation vortices, which are induced by the gradual adverse pressure gradient near the leading edge. Therefore, the wall-model failure directly translated into an incorrect estimate of the momentum flux and thus a different boundary layer thickening. This interesting feature is presented in Fig. 6, which shows that the wall-model simulation cannot capture the leading edge bubble properly. In fact, the laminar part of the boundary layer is treated with a wall-model, which enhances the friction and hence leads to an artificial growth of the boundary layer. Therefore, because the turbulent boundary layer has a weaker resistance against separation, the separation occurs earlier. For a very small recirculation region as in the current cases, the wall-model pushes the flow to recover a turbulent boundary layer. This numerical behavior is problematic and is the cause of the very small size of the recirculation region. However, the vortical structures at the trailing edge and the wake are similar to the reference WRLES case; see Figs. 4 and 5.

**Figure 7:**
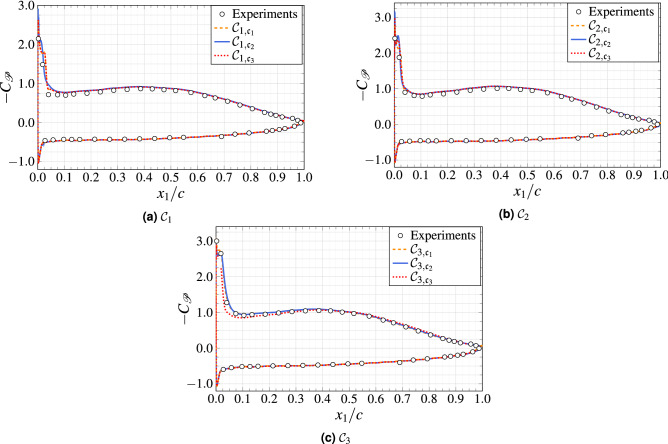
Comparison of the time-averaged pressure coefficient distribution.

### Mean flow profiles

In Fig. 7, we present the time- and spanwise-averaged pressure coefficient, $C_{{\rm {\mathscr{P}}}}$, computed as

8 $$\begin{aligned} C_{{\mathscr{P}}}= \frac{\langle{\mathscr{P}}\rangle -{\mathscr{P}}_\infty }{\frac{1}{2}\rho _\infty{\mathscr{U}}_\infty ^2}, \end{aligned}$$

where $\langle {\rm P}\rangle$ is the time- and spanwise-averaged wall pressure and ${\mathscr{P}}_\infty$ is the reference pressure taken at the outlet boundary. The whole pressure side of the blade is subjected to a favorable pressure gradient and the boundary layer is completely attached. On the suction side, the separation bubble creates a strong pressure drop close to the leading edge and large fluctuations of the wall shear stress are observed in the reattachment region. Downstream of the separation bubble, the mean pressure increases slightly up to mid-chord, then an adverse pressure gradient is observed up to the trailing edge. The mean flow features described in the previous section are recovered quantitatively with the numerical results, with a slight mismatch for $C_{{\mathscr{P}}}$ at the beginning of the separation zone. The results obtained with the WMLES approach are very close to the results of the reference WRLES configuration. However, note that the discrepancies observed at the leading edge are not surprising since our numerical simulations do not account for the jet shear layers and the possible introduction of the free-stream turbulence that are present in the experiments^30^. We observe a small difference in the leading edge region between the WRLES and WMLES cases. However, we found very good agreement between the WMLES calculation and the reference WRLES for the surface pressure coefficient near the trailing edge region, and we found significant improvements between the iso-resolution configurations ${{\mathcal{C}}}_{3,{\mathfrak{c}}_2}$ (with the wall-model) and ${{\mathcal{C}}}_{3,{\mathfrak{c}}_3}$ (without the wall-model). Therefore, we can conclude that the WMLES approach is particularly suitable for the estimation of the leading edge turbulent boundary layer characteristics. At this stage, we must acknowledge that the accurate prediction of $C_{{\mathscr{P}}}$ is a common finding in most external aerodynamics calculations. This result is most probably attributed to the outer-layer nature of the mean pressure, which appears to be less sensitive to the flow details of the near-wall turbulence^31^.

**Figure 8:**
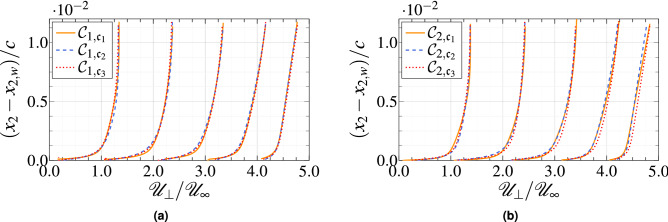
Mean streamwise velocity (${\mathscr{U}}_{\bot }/{\mathscr{U}}_{\infty }$) as a function of wall-normal distance at $x_1/c=0.1$, 0.3, 0.5, 0.7 and 0.9. Each plot is separated by a horizontal offset of 1.0.

The profiles of the mean velocity normal to wall ${\mathscr{U}}_{\bot }$ are shown in Fig. 8 for the cases ${{\mathcal{C}}}_1$ and ${{\mathcal{C}}}_2$. The profiles of the case ${{\mathcal{C}}}_3$ are roughly similar to the case ${{\mathcal{C}}}_2$; unless otherwise stated, only the results of ${{\mathcal{C}}}_2$ are shown. The velocity profiles show almost no deviation compared to the reference WRLES. The relative errors of the three mean velocities are less than 2% for the five locations considered. The turbulent boundary layer over the airfoil is about 10–20 mm thick, which, with our grid resolution, results in roughly 20–40 points per boundary later thickness, $\delta$. Assuming that the flow at a given station can be approximated by a local canonical zero-pressure-gradient turbulent boundary layer, the expected error in the mean velocities can be estimated as $\varepsilon _m=0.4\Delta /\delta$ (with $\Delta$ an isotropic grid size), which yields values of 2% for the current grid resolutions. The previous estimation is strictly valid for the zero-pressure-gradient turbulent boundary layer and, as such, it should be understood only as representative of the errors in complex geometries. However, this estimation provides a useful reference for the expected performance of WMLES in the absence of 3D and non-equilibrium effects for the current grid resolutions.

**Figure 9:**
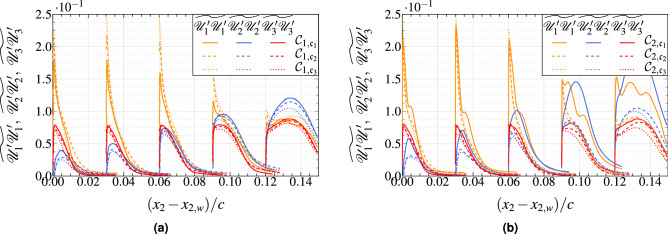
Resolved Reynolds shear stress profiles as a function of wall-normal distance at $x_1/c=0.1$, 0.3, 0.5, 0.7 and 0.9. Each plot is separated by a horizontal offset of 0.03.

In Fig. 9, the Reynolds stress component at five different locations are also plotted. The trend followed by all the stress components is correctly captured, although their magnitudes are systematically underpredicted by $\sim \, 18\%$. In fact, they present a slight deviation consistent with the modeling approach, which supports a limited fraction of the turbulent fluctuations. This behavior is typical for WMLES, which usually tends to overestimate the streamwise fluctuations while underestimating the transverse components. This is a direct effect of the grid resolution; the turbulent flow cannot carry identical turbulent structures in the near-wall region, which affects the Reynolds stress structures. However, the difference in the Reynolds stresses is mainly localized in a narrow region very close to the wall. Thus, it should not significantly affect the pressure fluctuations whose maximum value is located further away from the solid wall. The prediction capability of the WMLES reduces close to the trailing edge for the streamwise component of Reynolds stress. Note that at iso-resolution, the resolved Reynolds stress computed by WMLES are in good agreement with the reference WRLES simulations compared to the case where the wall-model is not activated.

**Figure 10:**
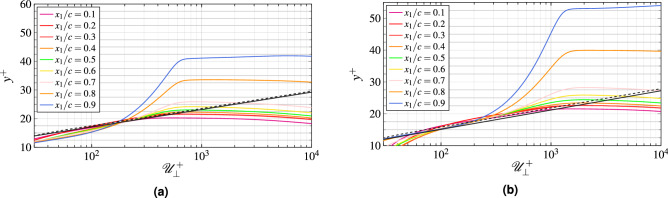
Profiles of the mean wall tangential velocity along wall-normal distance. The dashed line corresponds to the Reichardt law of the wall and the solid line to the classical law of the wall. The case ${{\mathcal{C}}}_3$ shows the same behavior and thus, for brevity, is not plotted.

In Fig. 10, the mean wall-parallel velocity profiles on the suction side are presented in log scale. The most basic wall-models are based on analytical laws that provide a direct link between the velocity normal to the wall ${\mathscr{U}}_{\bot }$ at a certain wall distance $x_2$ and the wall-shear stress $\tau _w=\rho{\mathscr{U}}_\tau ^2$. The most widely used law is the Reichardt law-of-the-wall^32^:

9 $$\begin{aligned}{\mathscr{U}}_{\bot }^+=\frac{1}{\kappa }\ln (1+y^+\kappa )+ \left( C-\frac{1}{\kappa }\ln (\kappa )\right) \left( 1-e^{-\frac{y^+}{11}}- \frac{y^+}{11}e^{-\frac{y^+}{11}}\right) , \end{aligned}$$

with ${\mathscr{U}}_{\bot }^+={\mathscr{U}}_{\bot }/{\mathscr{U}}_\tau$ and $y^+=y{\mathscr{U}}_\tau /\nu$. Although this law is dedicated to equilibrium flows, it should be applicable to out-of-equilibrium flows as long as the input position is chosen below the end of the log-layer region. The constant $\kappa$ and *C* are the same than those of the typical von Kármán classical law of the wall^33^

10 $$\begin{aligned}{\mathscr{U}}_{\bot }^+=\frac{1}{\kappa }\ln (y^+)+C. \end{aligned}$$

Both analytical approaches are compared in Fig. 10. Notice that the wall-parallel velocity component profiles show a fast increasing boundary layer thickness after 50% of the chord, due to a strong adverse pressure gradient on the blade. At $x_1/c=0.9$, the flow is nearly at the edge of the separation, but is still attached ($\partial{\mathscr{P}}/\partial x_2$ is positive). All WMLES profiles show quite a large log-region, which collapse on the Reichardt law-of-the-wall for $y^+\le 300$. If the wall-model input is chosen below that threshold, the equilibrium wall-model in this study appears to be sufficient. Nagib and Chauhan^34^ have emphasized the non-universality of the Kármán constant $\kappa$ through various experiments on canonical flows and showed that FPG leads to higher values of $\kappa$ while adverse pressure gradient gives lower $\kappa$. They also showed that the constant *C* can be negative. Therefore, the comparability between our results and the two proposed analytical approaches could be improved by finding an optimal choice of $\kappa$ and *C* values.

**Figure 11:**
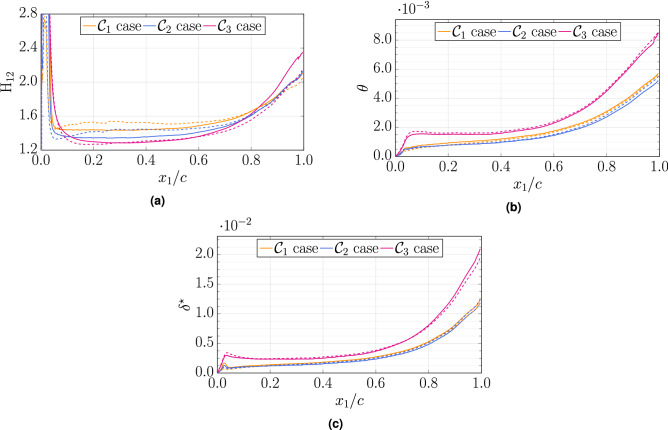
Variations of shape factor ${\rm H}_{12}$, momentum thickness $\theta$, and displacement thickness $\delta ^\star$ with chordwise distance $x_1/c$ between WRLES computations (solid lines, cases ${{\mathcal{C}}}_{*,{\mathfrak{c}}_1}$) and WMLES computations (dashed lines, cases ${{\mathcal{C}}}_{*,{\mathfrak{c}}_2}$).

In Fig. 11, we show the variation of the shape factor (${\rm H}_{12}=\delta ^{\star }/\theta$), the momentum thickness $\theta$, and the displacement thickness $\delta ^\star$ against the chordwise distance $x_1/c$ along the aerofoil upper surface. In the case of aerofoil flows, the traditional definition of boundary layer parameters as used for flat plates must be adapted to be consistent with the spatially varying potential flow. In the present study, the definitions used for the displacement thickness $\delta ^\star$ and the momentum thickness $\theta$ follow the recommendation of Wagner et al.^35^. Near the leading edge, the flow is laminar and the shape factor is approximately 2.6 for ${{\mathcal{C}}}_{*,{\mathfrak{c}}_1}$ cases, which is a typical value of the Blasius profile, while the ${{\mathcal{C}}}_{*,{\mathfrak{c}}_2}$ cases feature a higher value $\sim 2.9$ (zoom not shown). The displacement thickness reaches a local maximum in the region of the separation bubble while the momentum thickness is nearly zero, which results in a very large shape factor. Downstream of the reattachment region ($x_1/c\simeq 0.03$ for all cases), the shape factor decreases abruptly, and then close to the mid-chord where there is close to a zero pressure gradient, a plateau is observed at $\sim 1.5$ for ${{\mathcal{C}}}_1$, 1.35 for ${{\mathcal{C}}}_2$ and $\sim 1.28$ for ${{\mathcal{C}}}_3$, which is smaller than the typical value of the Klebanoff profile ${\rm H}_{12}\simeq 1.4$ observed for turbulent boundary layers). Along the second half of the chord, both the two thicknesses and the shape factor increase because of the adverse pressure gradient. A larger difference is observed between ${{\mathcal{C}}}_2$ and ${{\mathcal{C}}}_3$. Those differences depend both on the Reynolds number and the pressure gradient^36^.

### Acoustic field

**Figure 12:**
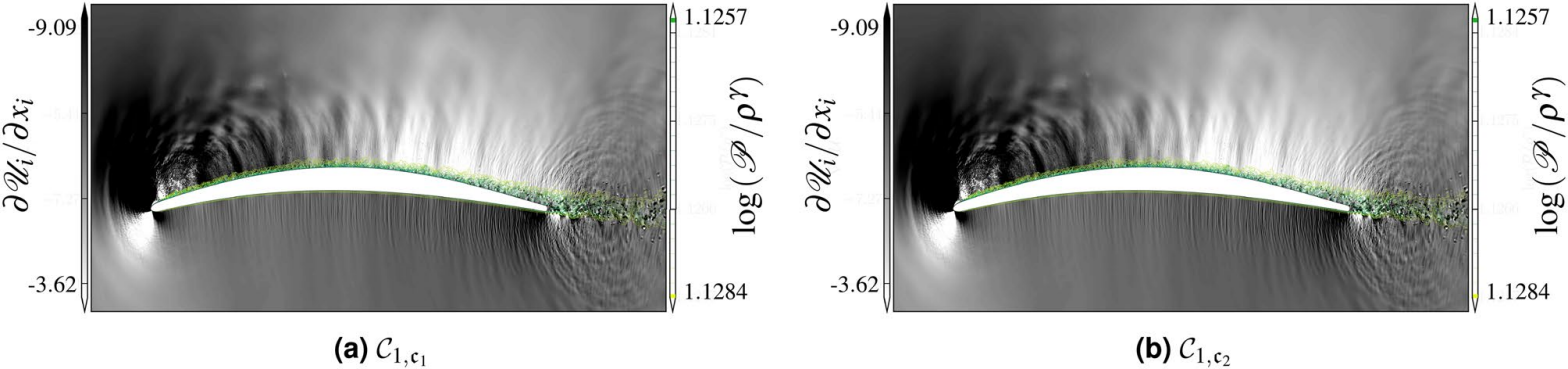
Instantaneous field of dilatation rate fluctuations and iso-entropy contours for the ${{\mathcal{C}}}_1$.

**Figure 13:**
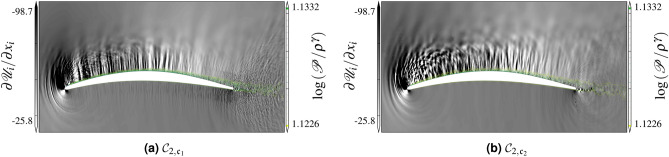
Instantaneous field of dilatation rate fluctuations and iso-entropy contours for the ${{\mathcal{C}}}_2$.

Analyzing the divergence of the velocity field is particularly useful to delineate the sources and the strength of the acoustic waves in the flow field. An instantaneous picture of the field of $\nabla \cdot{\mathscr{U}}$ on the midspan plane is shown in Figs. 12 and 13 along with contours of iso-entropy $s=\log ({\mathscr{P}}/\rho ^\gamma )$. The ${{\mathcal{C}}}_3$ simulation is found to be qualitatively similar to the ${{\mathcal{C}}}_2$ simulation. Two major acoustic source regions can be visually identified: the trailing edge and the transition/reattachment zones on the upper surface. Contrary to the study of Wu et al.^37^, the near wake does not appear to contribute significantly to the self-noise. The transition/reattachment noise source appears to be even stronger than the trailing edge noise for this flow configuration. This is likely caused by the high local Mach number due to the strong flow acceleration near the leading edge where the blade curvature is higher. An additional sound source is visible near the leading edge on the suction side of the lifting surface. This sound source is mainly caused by the massively accelerated vortex structures near the leading edge^38^. The transition/reattachment noise source radiates mostly downstream as it is shielded by the upper blade surface (with only a slight diffraction at the leading edge), whereas the trailing edge source shows an anti-symmetric pattern on each side of the blade, as expected from classical trailing edge noise theories^39^. Note the existence of a noise source near the trailing edge. The vortical structures in the turbulent boundary layer pass the trailing edge and generate sound. Visually, the WMLES approach seems to reproduce a qualitatively similar acoustic source field to the reference WRLES simulations. For the higher Mach number cases ${\mathscr{C}}_2$–${\mathscr{C}}_3$, we notice a stronger radiation in the presence of the separating shear layer, which generate additional noise sources and affect the propagation of acoustic waves upstream by the refraction through the separated shear layer. The secondary source, at higher frequency (closer wave fringes), appears less on the suction side, near the leading edge, close to the reattachment point of the recirculation bubble when using WMLES, which results from the fact that the current equilibrium WMLES does not show properly the laminar separation.

### Frequency spectra of surface pressure fluctuations

**Figure 14:**
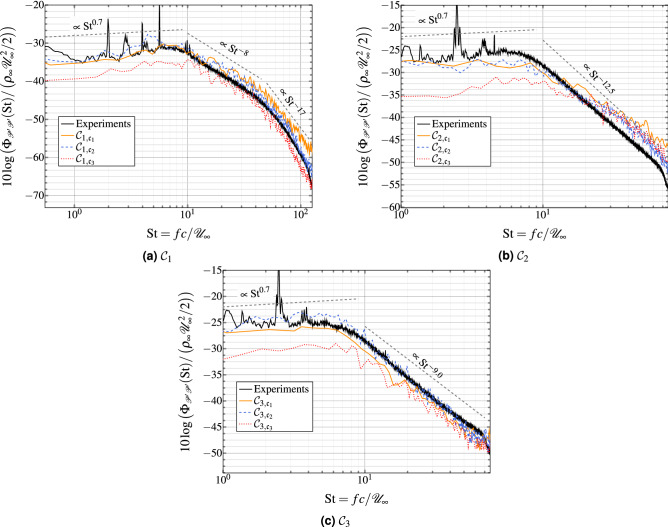
Wall pressure power spectral density (pressure side) at $x_1/c=0.976$.

Here we assess the capability of wall-modeled LES to predict surface pressure fluctuations on the suction side of the airfoil surface at $x_1/c=0.976$, as shown in Fig. 14. The frequency spectra of pressure fluctuations (PSD) as well as the cross-spectral and auto-spectra have been estimated numerically based on the Welch method of periodogram^40^, which minimizes the variance of the PSDs estimator^41^. The overall pressure signals from our computations, extracted from probes that are placed in the first wall-normal cell along the airfoil, are subdivided into ${\mathscr{N}}_{\rm s}/16$ sub-blocks, where ${\mathscr{N}}_{\rm s}$ represents the number of pressure recordings of each case. We adopt the Welch method combined with a Hanning window throughout the spectral analysis while using a FFT. The overlapping of the sub-blocks is set to 50%. The frequency spectrum is then obtained by averaging the periodograms of all the sub-blocks. The normalized sampling frequency is set to 3,000, i.e., we collect 3,000 pressure signals per each probe and convective time scale. The PSD is plotted as a function of the streamwise location and the normalized frequency (or Strouhal number ${{\rm St}}=fc/{\mathscr{U}}_{\infty }$). The trailing edge noise displays a low frequency broadband spectrum with most of the energy of the signal below ${{\rm St}}=9$. The PSD of each case features noticeable differences, as indicated by the slopes that are superimposed onto each dimensionless PSD. In general, the WMLES is found to underpredict the spectral level of the experimental data by 2–4 dBs in the low to intermediate range of frequency. The high range of frequency of the experimental pressure spectra is more accurately captured by the WMLES compared the reference WRLES’s. The most striking finding in this figure is that at iso-resolution, WMLES cases are in better agreement than the cases without the wall-model, mainly at the low frequency range. Overall, all of the spectra in Fig. 14 demonstrate that the broadband content is predicted reasonably well by the WMLES, which provides a good baseline study to assess and quantify noise prediction. The tonal peaks that arise from the experimental spectra on Fig. 14 are attributable to acoustically untreated duct models of the wind-tunnel facility. Moreover, the observed disparities at low Strouhal number may be partly caused by the installation effects of the experiment that are not accounted for in the numerical simulations, but also to a lack of statistical convergence of the WRLES spectra as a result of the time series being short.

**Figure 15:**
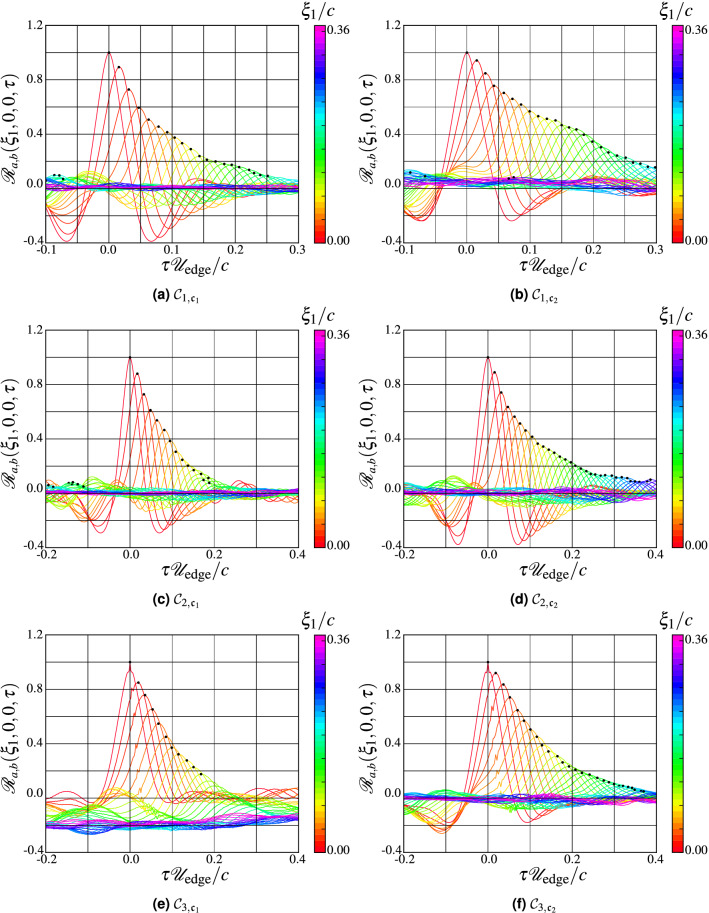
Cross-correlation functions of the simulated surface pressure fluctuations on the controlled-diffusion airfoil at suction side computed for different normalized streamwise separations $\xi _1/c$.

The wall-pressure statistics computed from the time-domain analysis are deduced from the temporal pressure cross-correlation calculated as

11 $$\begin{aligned}{\mathscr{R}}_{{a,b}}(\xi _1,0,\xi _3,\tau )=\langle{\mathscr{P}}'(x_1,0,x_3,t){\mathscr{P}}'(x_1+\xi _1,0,x_3+\xi _3,t+\tau )\rangle , \end{aligned}$$

where ${\mathscr{P}}^\prime$ is the surface pressure fluctuations, *a* and *b* represent two arbitrary points on the airfoil separated by distance $\xi _1$, $\xi _3$ in the streamwise and spanwise directions, respectively. The brackets $\langle \cdot \rangle$ indicate an ensemble average. The time delay between two signals $\tau$ is normalized on the chord *c*, and the inflow velocity ${\mathscr{U}}_\infty$. The effect of streamwise separation is depicted in Fig. 15. The cross-correlation ${\mathscr{R}}_{{a,b}}(\xi _1,0,0,\tau )$ is computed using the probe located at $x_1/c=0.975$ as a reference for the computation of correlation with upstream probes that are separated by $\xi _1$. We note the diminution of the correlation between the two signals with increasing separation between the signals. The decreasing peak of cross-correlation is due to the change in the pressure signature in the turbulent boundary layer, since the greater the distance between the probes, the greater the distance over which the structures can change and evolve, which yields a lower correlation between the pressure signals. For the WMLES cases, we notice that the pressure signals are correlated over a larger separation distance, and thus for a higher time delay relative to the reference WRLES configurations. This can be explained by the fact that the structures evolve less rapidly when using the wall model, i.e., the structures are constrained by using the wall-model, leading to much lower correlation value of ${\mathscr{R}}_{{a,b}}(\xi _1,0,0,\tau )$.

Using the function ${\mathscr{R}}_{{a,b}}(\xi _1,0,0,\tau )$, it is possible to estimate the value of convection velocity of the vortical structures ${\mathscr{U}}_c(\xi _1)$ as a function of the separation distance $\xi _1$ in the streamwise direction between probes as

12 $$\begin{aligned} \frac{{\mathscr{U}}_c(\xi _1)}{{\mathscr{U}}_{{\rm edge}}}= \frac{\xi _1/\delta ^\star }{\left[ \tau{\mathscr{U}}_{{\rm edge}}/\delta ^\star \right] _{\max }}, \end{aligned}$$

where $\left[ \tau{\mathscr{U}}_{{\rm edge}}/\delta ^\star \right] _{\max }$ represents the time lag, which corresponds to the maximum of the cross-correlation ${\mathscr{R}}_{{a,b}}(\xi _1,0,0,\tau )$ between two pressure signals separated by a normalized distance $\xi _1/\delta ^\star$.

**Figure 16:**
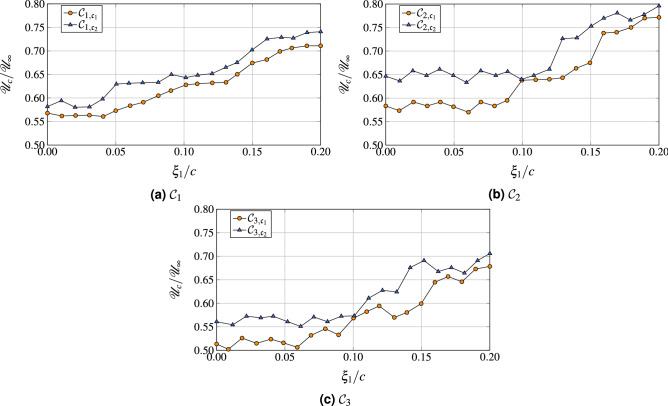
Convection velocity as a function of $\xi _1$, normalized by the chord. Reference probes used for the longitudinal spacing $\xi _1$ is $x_{{\rm ref}}=0.978$.

In Fig. 16, the normalized convection velocity is extracted as a function of the longitudinal separation $\xi _1$, taken as the reference probe located at $x_1/c=0.978$, for both the reference WRLES and WMLES computation. The convective velocity ranges from $0.55\cdot{\mathscr{U}}_\infty$ to $0.80\cdot{\mathscr{U}}_\infty$ with an apparent dependency on the Reynolds number or angle of attack. WMLES seems to cause a higher streamwise convective velocity ${\mathscr{U}}_c$ with respect to the baseline WRLES configuration.

### Cross-spectral density and coherence

One of the main quantities generally used to describe wall-pressure statistics is the cross-spectral density in the spanwise direction, which is defined by

13 $$\begin{aligned} \Gamma _{{{\text{P}}_{a}}{{\textsf{P}}_{b}}}(\xi _{1},0,\xi _{3},\omega ) =\langle \widehat{\mathscr{P}}'^{*}(x_{1},0,x_{3},\omega ), \widehat{\mathscr{P}}'(x_{1}+\xi _{1},0,x_{3}+\xi _{3},\omega )\rangle , \end{aligned}$$

where $\widehat{\mathscr{P}'}$ is the time FFT transform of the fluctuated wall pressure. The complex conjugate is denoted by $\cdot ^{*}$. According to the coherence function, which is used to indicate the strength of the relation between the pressure fluctuations at two separate locations as the frequency varies, as the disturbed flow is convected in the streamwise or in the spanwise direction, it is possible to express the cross-spectrum in non-dimensional form. Hence, the coherence function can be expressed as

14 $$\begin{aligned}{\gamma }_{{a,b}}(\xi _{1},0,\xi _{3},\omega ) =\frac{|\Gamma _{{{\textsf{P}}_{a}}{{\textsf{P}}_{b}}}(\xi _{1},0,\xi _{3},\omega )|}{\sqrt{\Gamma _{{{\textsf{P}}_{a}}{{\textsf{P}}_{a}}}(\omega ) \Gamma _{{{\textsf{P}}_{b}}{{\textsf{P}}_{b}}}(\omega )}}, \end{aligned}$$

where $\Gamma _{{{\textsf{P}}_{a}}{{\textsf{P}}_{a}}}$, $\Gamma _{{{\textsf{P}}_{b}}{{\textsf{P}}_{b}}}$ are the pressure auto-spectra at the two positions *a* and *b*, respectively. The different analytical expression coherence functions proposed in the literature are a function of the angular frequency $\omega$ (or equivalently the Strouhal number ${\rm St}=\omega \delta /u_\tau$), and normalized with respect to $\xi _3$ and ${\mathscr{U}}_c$, which are computed from the cross-correlation functions.

Despite its simplicity, the Corcos model^42^ is still extensively used in many practical applications, since it provides a simple analytical form and clear physical significance. This model is based on the assumption that the correlation lengths in both the streamwise and spanwise directions are statistically independent, and thus the coherence takes the form

15 $$\begin{aligned}{\gamma }_{{a,b}}(\xi _1,0,\xi _3,\omega )= e^{-\alpha _1k_c|\xi _1|}e^{-\alpha _3k_c|\xi _3|}e^{-\alpha _1k_c\xi _1}, \end{aligned}$$

where $k_c=\omega /U_c$ is the convective wavenumber. In the formulation, $\alpha _1$ and $\alpha _3$ are constants that measure the loss of coherence in the streamwise and spanwise directions, for which different values can be found in the literature.

A second semi-empirical model, which is an extension of the Corcos model, has been proposed by Efimtsov^43^ and improved by Salze et al.^44^. This model explicitly considers the compressibility effects by introducing a number of additional tunable constants. The coherence function is expressed for this model, according to the multiplicative hypothesis (cf. (15)), as

16 $$\begin{aligned}{\gamma }_{{a,b}}(\xi _1,0,\xi _3,\omega )= e^{-|\xi _1|/\Lambda _1}e^{-|\xi _3|/\Lambda _3}e^{-\alpha _1k_c\xi _1}, \end{aligned}$$

where $\Lambda _1$ and $\Lambda _3$ are the correlation lengths in the streamwise ($\Lambda _1={\mathscr{U}}_c/\omega \alpha _1$) and spanwise direction ($\Lambda _3={\mathscr{U}}_c/\omega \alpha _3$), respectively. For a free stream Mach number below 0.75, the spanwise correlation length is modeled as follows^44^

17 $$\begin{aligned} \frac{\Lambda _3}{\delta }=\left[ \left( a_4\frac{\omega \xi _3}{{\mathscr{U}}_c}\frac{\delta }{\xi _3}\right) ^2+ \frac{\left( a_5\frac{u_\tau }{{\mathscr{U}}_c}\right) ^2}{\left( \frac{\omega \xi _3}{{\mathscr{U}}_c} \frac{\delta }{\xi _3}\right) ^2+\left( \frac{a_5}{a_6}\frac{u_\tau }{{\mathscr{U}}_c}\right) ^2} \right] ^{-1/2}, \end{aligned}$$

where the empirical parameters $a_{4,5,6}$ are adjustable through proper fit of experimental or numerical data. According to Palumbo^45^, the Efimtsov–Salze’s constant $a_4$ constrains the amplitude of mid- to high-frequency coherence lengths, similar to the constants in the Corcos model. The frequency at which the Efimtsov–Salze model breaks away from the Corcos model is governed by the parameter $a_5$, while the low-frequency roll off is controlled by the parameter $a_6$.

**Figure 17:**
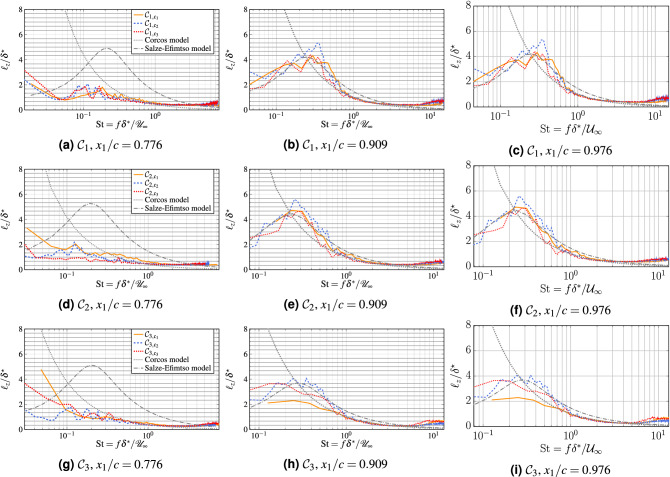
Spanwise coherence length at different locations close to the trailing edge for the three cases.

The major limitations of the Salze–Efimtsov model are the tunable constants $a_{4,5,6}$, which generally depend on the Mach and Reynolds numbers, as well as on the separation distance. These parameters are estimated in this study at $x_1/c=0.976$ by fitting the coherence data points obtained from the reference WRLES configurations, by means of a nonlinear least-squares optimization procedure. The coefficients thus obtained in the range of Mach numbers considered are $a_4=0.47$, $a_5=17.80$ and $a_6=1.0$. We depict in Fig. 17 the spanwise coherence length at different locations close to the trailing edge for the three cases. We see that the collapse of the curves for both the Corcos or Salze–Efimtsov models is achieved at high frequencies for all the tested computations at $x_1/c=0.976$. We obtain less satisfactory agreement between the theoretical predictions and the numerical data far upstream $x_1/c=0.976$ (for which the parameters of Salze–Efimtsov model are computed). Nevertheless, by comparing spectra obtained for the two locations $x_1/c=0.909$ and $x_1/c=0.976$ , it can be concluded that very close to the trailing edge, the wall-pressure statistics are well established and almost stationary justifying the use of radiation models based on a wall-pressure statistics at a single point close to the trailing-edge^46,47^. The Salze–Efimtsov model predicts the peak in coherence lengths and compares reasonably well to the Corcos model. The Salze–Efimtsov also achieves a much better agreement with the present simulated dataset at lower frequencies. The lack of agreement for the Corcos model at low frequencies is predicted, since the Corcos model is not suited for this range of frequencies.

Moreover, good agreement between the predictions of the Salze–Efimtsov model and the numerical data is obtained in all cases. Unlike the Corcos approximation, the Salze–Efimtsov model is able to provide a good agreement in the low-frequency range of the coherence functions, especially at $x_1/c=0.976$ for all the matrix computation. We note that the comparison of coherence length distribution at ${{\rm Re}}=2.29\times 10^6$ between both models and the simulated results is better than the case ${{\rm Re}}=8.30\times 10^5$, suggesting a Reynolds number dependence of the shape of these models. We can also see that the WMLES curves fit the reference WRLES computations reasonably well, regardless of the studied case.

## Conclusion

The prohibitive computational cost of resolving the inner region of turbulent boundary layers prevents using the WRLES approach to study high-Reynolds number turbulent flows in complex geometries. However, using the wall-modeled LES approach can reduce this computational cost. The major objective of this work was to evaluate the feasibility and accuracy of the WMLES approach for the prediction of broadband noise generated by a controlled-diffusion blade. To this end, we carried out detailed comparisons between the results obtained with the equilibrium WMLES approach, experimental measurements, and the wall-resolved LES reference solutions. LES with wall-modeling accurately predicted the pressure coefficient distribution, velocity statistics (including the mean velocity), and the trace of Reynolds tensor components. We also found that, for the wall-modeled LES cases, the instantaneous flow structures resembled those observed in the reference wall-resolved LES, except near the leading edge region. In addition, the boundary layer thickness, displacement thickness, and momentum thickness along the airfoil chord showed a convergent behavior towards those obtained using the wall-resolved LES. Surface pressure fluctuations, which act as the sources of the broadband noise at the far-field, were also extracted in the form of surface pressure spectral densities along the airfoil chord for both the wall-modeled LES and wall-resolved LES calculations. Our results indicate that the pressure spectral density profiles from the wall-modeled LES compare well with the experimental profiles. Furthermore, we found that wall-modeled LES is more comparable with the experimental data in the high-frequency region than the WRLES reference case. The convection velocity showed an increase towards an asymptotic value as the separation distance, $\xi _1$, increased, and we found that the WMLES results overestimated the WRLES computations. We compared the spanwise correlation length to the Corcos^42^ model, which assumes a frequency-independent correlation length, and the Salze–Efimtsov model^44^, which, in contrast, introduces a frequency dependence. We found both WMLES and WRLES computations compare well with both models over the higher range of frequency, which accounted reasonably well for the effects of the pressure gradients. Nevertheless, the Salze–Efimtsov model was in better agreement with the computational results for the lower range of frequency, but two parameters needed to be tuned. Overall, the equilibrium WMLES presents an interesting approach to (i) investigate in more detail the turbulent pressure field induced by turbulent boundary layer over the controlled-diffusion airfoil, and (ii) advance the development of new versions of trailing edge noise models on coarser grids, which is needed for fast simulations in industrial design.
